# Laparoscopic treatment for appendicitis during pregnancy: Retrospective cohort study

**DOI:** 10.1016/j.amsu.2021.102668

**Published:** 2021-08-05

**Authors:** Carina Chwat, Marcelo Terres, Mauro Ramírez Duarte, Diego Valli, Flavia Alexandre, Guillermo Rosato, Gustavo Lemme

**Affiliations:** Hospital Universitario Austral, Colorrectal Unit, General Surgery Department, Presidente Perón 1500; Derqui, Buenos Aires, Argentina

**Keywords:** Acute appendicitis, Pregnancy, Complications

## Abstract

**Background:**

Acute appendicitis is the most frequent non-obstetric surgical emergency during pregnancy. The benefits of laparoscopy during pregnancy are well known, but complications can occur, and these can affect both the mother and/or the foetus.

We present results of laparoscopic surgical treatment of acute appendicitis in pregnant women, analysing the occurrence of adverse postoperative, obstetric and foetal outcomes and reviewing literature.

**Materials and methods:**

Retrospective observational study on pregnant women with a preoperative diagnosis of acute appendicitis.

**Results:**

n = 63, mean age 28.4 years, average gestational age of 17.7 weeks (3–30 weeks). 6.4 % exploratory laparoscopies, 92 % laparoscopic appendectomies and one right colectomy were performed. Conversion rate was 3.2 %. When symptoms begun within 48 hours prior to surgery, a perforated appendicitis was found in 11 %; whereas when the time from symptom onset to surgery was greater than or equal to 48 hours, it was evident in 31 % of the cases (p 0.008). The only independent variable associated with the presence of postoperative complications was symptom duration prior to surgery greater than or equal to 48 hours (OR 4.8; 95 % CI 1.1–16.2; p 0.04). Seven minor and 2 mayor postoperative complications were observed. Patients with complications spent, on average, twice as many days hospitalized (p < 0.001); and had 8 times more risk of preterm delivery (p 0.03). Obstetric complications were more frequent in pregnant women operated during the first trimester. Foetal mortality was 1.6 %.

**Conclusion:**

Surgical morbidity of acute appendicitis in pregnant women is linked to the delay in the diagnosis and treatment of the inflammatory condition. Laparoscopic appendectomy during pregnancy is not exempt from postoperative, obstetric and foetal complications. It is necessary to standardize the definitions of “complication” in order to collate reliably the outcomes presented in the literature.

## Highlights

• Appendicitis is the most frequent non-obstetric surgical emergency during pregnancy.
• Complications of laparoscopic surgery can affect the mother and/or the foetus.
• Symptoms' duration prior to surgery is associated with presence of complications.
• Complicated patients have 8 times more risk of preterm delivery.

## 1 Introduction

Acute abdomen in pregnancy (AAP) represents a diagnostic and therapeutic challenge due to its multiple obstetric and non-obstetric causes, as well as the anatomical and physiological changes that occur during pregnancy, alterations in usual laboratory parameters and the reluctance to use diagnostic modalities such as radiography and tomography [1,2].

Accurate and timely diagnosis and treatment decrease maternal and foetal morbidity and mortality [3].

Acute appendicitis is the most frequent non-obstetric surgical emergency during pregnancy, with an incidence between 0.04 % and 0.2 % [4,5]. Although it can occur at any time during pregnancy, the incidence is higher in the second trimester. It is the most common cause of non-obstetric surgical procedures performed during pregnancy, representing 25 % [6].

Other non-obstetric causes of AAP include cholecystitis, ovarian torsion, splenic disorders, symptomatic hernias, complications of inflammatory bowel diseases, acute pancreatitis, intestinal obstruction, and trauma [7].

In the past, laparoscopy was contraindicated due to the risk of uterine injury with surgical instruments, greater technical difficulty due to a reduced working space because of the uterus, the concern for foetal acidosis secondary to insufflation with carbon dioxide and the decrease in maternal venous return owing to intra-abdominal pressure increase because of pneumoperitoneum. Currently, numerous studies have presented laparoscopy as a feasible, safe and effective therapeutic option throughout pregnancy [[8], [9], [10]].

The benefits of laparoscopy during pregnancy include less uterine manipulation, less postoperative ileus, decrease in foetal respiratory depression due to lower narcotic requirements for pain management, lower incidence of wound complications and thromboembolic events, shorter hospital length-of-stay and a prompt return to normal activity [[11], [12], [13]]. Nonetheless, complications can occur, and these can affect both the mother and/or the foetus.

We present our results of laparoscopic surgical treatment of acute appendicitis in pregnant women, analysing the occurrence of adverse postoperative, obstetric and foetal outcomes and reviewing literature.

## 2 Methods

A retrospective cohort single-centred study was carried out, using computerized medical records’ information of pregnant patients of 17 or more years of age with AAP admitted to our institution between September 2005 and July 2020. The cohort was assembled in a consecutive order, and all patients were included.

Construction and analysis of the database and study protocol were authorized by the Institutional Evaluation Committee under N° B20-010 and P20-070, respectively, and registered at Clinicaltrials.gov under the Identifier: NCT04753502 (https://clinicaltrials.gov/show/NCT04753502).

Assuming a 3: 1 relationship between surgery within 48 h of symptom onset and surgery after 48 h, we have an 80 % power to find a difference in postoperative morbidity between groups of 30 % with error type I of 0.05.

Analysed variables included: demographic characteristics, gestational age, clinical presentation, symptom duration from onset until surgical resolution, complementary studies, ASA score (Classification of the American Society of Anaesthesiology), intraoperative findings, type of surgery performed, intra and postoperative complications, length of stay, readmissions, use of tocolytics, preterm delivery, birth weight, Apgar score, maternal and foetal mortality, and obstetric and perinatological complications in pregnant patients with a preoperative diagnosis of acute appendicitis.

Any surgery in which the exploratory laparoscopy did not reveal intra-abdominal pathology and the appendix was macroscopically and microscopically normal was classified as a “negative laparoscopy”. An “intraoperative complication” was defined as any unexpected intraoperative event, excluding conversion to conventional surgery, which was analysed as an independent event. A “postoperative complication” was defined as any deviation from the usual postoperative course within 30 days of surgery. An “obstetric complication” was one that occurred from the appendectomy until the end of the pregnancy, including foetal death and excluding preterm delivery. Spontaneous abortion and foetal demise were included within the same “foetal loss” outcome. “Preterm labour” was considered to be deliveries or caesarean sections that occurred prior to the 37th week of gestation.

Data report was done in line with the STROCSS criteria [14].

For continuous variables, mean, standard deviation and/or minimum and maximum, or median and interquartile interval (IQR) were used, according to distribution. For categorical variables the number and corresponding percentages were reported. Continuous parameter comparisons were made using the test for independent samples or Wilcoxon-rank test; and when there were more than two groups, Anova or Kruskal Wallis were applied. For the comparison of categorical variables, Chi-square or Fisher's exact test were used, as appropriate. A p < 0.05 was considered statistically significant. Likewise, a multivariate analysis was performed for the presence of postoperative and obstetric complications, contemplating possible confounders.

Statistical analysis was performed with STATA 14.2 (StataCorp 2015. Stata Statistical Software: Release 14. College Station, TX: StataCorp LP).

### 2.1 Surgical procedure

The surgery was performed by the on-call surgeon or a senior resident under the surgeon's guidance, following the American Society of Gastrointestinal Endoscopic Surgeons' guidelines [15].

Continuous monitoring of carbon dioxide pressure at the end of expiration and antibiotic prophylaxis were implemented. Patients were placed in supine position, in Trendelenburg, lateralized 30° to the left, avoiding the compression of the inferior vena cava and improving the exposure of the ceco-appendicular area. Abdominal cavity entry was done using an open technique with placement of a Hasson trocar, adjusting incision height according to the trimester, anticipating uterine growth. The other two trocars (one 10 mm and the other 5 mm) were placed in such a way that a correct triangulation of the laparoscopic instruments was achieved to perform the procedure. The pressure within the abdominal cavity was kept below 12 mmHg. An exploratory laparoscopy was systematically performed to rule out other causes of AAP. The appendectomy technique was identical to that performed in non-pregnant patients. During the entire procedure, direct manipulation of the uterus was avoided. Uterine-inhibition was not systematically used.

An obstetric ultrasound was performed both prior and immediately postoperatively, and postoperative pneumatic compression boots or early ambulation were used as prophylaxis for postsurgical deep vein thrombosis.

## 3 Results

Within the study period, ninety pregnant women were treated for non-obstetric acute abdominal pain, of which 70 % (N = 63) had a preoperative diagnosis of acute appendicitis.

Mean age was 28.4 years, with a range of 17–47 years; and average gestational age of 17.7 weeks (3–30 weeks); 60.3 % (N = 38) were in the second trimester of pregnancy, 25.4 % (N = 16) in the first, and 14.3 % (N = 9) in the third trimester. All were singleton pregnancies and 75 % had no previous abdominal surgeries. A pregnant woman in the 16th week of pregnancy at the time of surgery presented situs inversus.

Acute abdominal pain was present in 100 % of women, 60.3 % (N = 38) had a Murphy chronology and 69.9 % (N = 39) had rebound pain. There was no difference in the presence of Murphy chronology according to trimester of pregnancy. Fever was present in 31.8 % and 76.2 % reported nausea and/or vomiting.

All patients diagnosed with acute appendicitis were approached laparoscopically: 6.4 % exploratory laparoscopies (N = 4), 92 % laparoscopic appendectomies (N = 58) and one right colectomy were performed. All procedures were completed laparoscopically, with the exception of 2 in which an appendicular plastron was found. For these, an open appendectomy via a classical McBurney incision was performed in one case, and a right hemicolectomy with anastomosis; in the other. The conversion rate was 3.2 %. There were no uterine lesions in the series. Median duration of surgery was 60 min (IQR 45–100 min), with no differences between trimesters.

The most frequent intraoperative finding was a phlegmonous appendicitis (69.8 %, N = 44). Gangrenous appendicitis was confirmed in 10 women (15.9 %), and in three, an appendicular plastron was found (4.8 %). Peritonitis was present in 56.1 % (N = 38) of patients at the time of surgery. In 6 patients, no intra-abdominal pathology was evident in the laparoscopic examination (9.5 % negative laparoscopies). Two of them underwent appendectomy and no additional procedures were carried out in the remaining four.

When the symptomatology begun within 48 h prior to surgery, a perforated appendicitis was found in 11 %; whereas when the time from symptom onset to surgery was greater than or equal to 48 h, it was evident in 31 % of the cases (p 0.008). Likewise, a statistically significant association was found between symptom duration and postoperative complications, present in 8.5 % of patients with less than 48 h of symptoms and in 45.5 % of those with more time from symptom onset to surgery (p 0.03). Furthermore, pre-operative symptom duration of 48 h or more was associated with a greater need for readmission (p 0.02).

One intraoperative complication was recorded. It was a 27-year-old patient, in the 22 nd week of pregnancy, who presented desaturation with pneumoperitoneum insufflation. It resolved immediately after lowering the insufflation pressures and relocating the endotracheal tube.

Median hospital stay was of 2 days (IQR 2–5 days). The only variable associated with the duration of hospitalization was the presence of postoperative complications (OR 2.9; 95 % CI 1.5–5.4; p 0.001).

There were no intra or postoperative complications, readmissions, reoperations, obstetric or perinatological complications in the patients in whom laparoscopy revealed a normal appendix.

Nine postoperative complications were observed, seven of them were minor complications (11.1 %) and 2 major complications (3.2 %), with no differences between trimesters of pregnancy. During postoperative hospitalization, 2 phlebitis, a urinary infection, a superficial umbilical incision wound infection and a Clostridium Difficile diarrhea were diagnosed. Phlebitis was treated with local measures, urinary tract infection and diarrhea with antibiotic therapy, and wound infection with wound lavage and oral antibiotic therapy.

Four pregnant women were readmitted within 30 days of surgery due to postoperative complications, representing a readmission rate of 6.35 %. A pregnant woman with diabetes was readmitted due to a urinary infection and poor glycemic control. Intravenous antibiotics, hemoglucotests and insulin corrections were indicated, and hospital discharge was granted after 4 days. Another patient presented with fever and was readmitted for evaluation. Phlebitis was diagnosed as the only cause of fever. It resolved with local measures and analgesia. Another patient on her 24th week of pregnancy was discharged 4 days afyer surgery with the drain in place, due to the presence of purulent discharge. Two days later, a 3x3-centimeter paracecal abscess secondary to an appendicular stump fistula was diagnosed by magnetic resonance imaging. Intravenous antibiotic treatment was indicated. The drain's discharge ceased on the 21st postoperative day, and subsequent ultrasound controls showed complete resolution of the abscess and fistula. The fourth patient presented abdominal terderness and rebound pain two days after discharge. Laparoscopic reexploration was performed. Right hemiabdominal and pelvic abscesses were found, proceeding to lavage and drainage, with adequate recovery and hospital discharge on the seventh day after surgery.

Patients with post-operative complications tended to be younger, have a higher white blood-cell count at admission, and a longer surgical time, compared to those who did not present complications. In the multivariate analysis, when adjusting for maternal age, trimester of pregnancy, ASA, intraoperative diagnosis and type of surgery, the only independent variable associated with the presence of postoperative complications was symptom duration prior to surgery greater than or equal to 48 h (OR 4.8; 95 % CI 1.1–16.2; p 0.04). Patients with complications spent, on average, twice as many days hospitalized (p < 0.001); and preterm delivery occurred in 25 %, while this occurred only in 3 % of the patients with no postoperative complications (p 0.03). No other differences were observed between the groups with respect to demographic variables, symptoms, intraoperative findings, tocolytic requirements, obstetric complications, birth weight or foetal loss (Table 1).

**Table 1:** Variables according to absence or presence of postoperative complications.

	POSTOPERATIVE COMPLICATION		p-value
VARIABLE	No (N = 54)	Yes (N = 9)	
Maternal age (years)			
	29 (6)	25 (6)	0.06
Gestational age (weeks)			
	18 (6)	18 (7)	0.87
Trimester of pregnancy			
1	14 (26 %) 32 (59 %) 8 (15 %)	2 (22 %) 6 (67 %) 1 (11 %)	0.91
2			
3			
Prior abdominal surgery			
No	37 (76 %)	5 (71 %)	0.82
Yes	12 (24 %)	2 (29 %)	
White blood-cell count (cells/mm^3^)			
	14,581 (3712)	17,189 (6113)	0.08
Murphy chronology			
No	21 (39 %) 33 (61 %)	4 (44 %) 5 (56 %)	0.75
Yes			
Rebound pain			
No	22 (41 %) 32 (59 %)	3 (33 %) 6 (67 %)	0.67
Yes			
Symptom duration prior to surgery			
<48 h	43 (80 %) 11 (20 %)	4 (44 %) 5 (56 %)	0.025
>48 h			
Type of Surgery			
Laparoscopy	4 (7 %) 49 (91 %) 1 (2 %)	0 (0 %) 9 (100 %) 0 (0 %)	0.64
Appendectomy			
Right Colectomy			
Intraoperative findings			
Normal appendix	6 (11 %) 39 (72 %) 7 (13 %) 2 (4 %)	0 (0 %) 5 (56 %) 3 (33 %) 1 (11 %)	0.24
Phlegmonous appendix			
Gangrenous appendix			
Appendicular plastron			
Conversion to open surgery			
No	52 (96 %) 2 (4 %)	9 (100 %) 0 (0 %)	0.56
Yes			
Peritonitis			
No	33 (61 %) 21 (39 %)	5 (56 %) 4 (44 %)	0.75
Yes			
Surgical time (minutes)			
	60 (45-60)	60 (60–100)	0.07
Tocolytic use			
No	49 (91 %) 5 (9 %)	9 (100 %) 0 (0 %)	0.34
Yes			
Intraoperative complication			
No	53 (98 %) 1 (2 %)	9 (100 %) 0 (0 %)	0.68
Yes			
Readmission			
No	54 (100 %) 0 (0 %)	5 (56 %) 4 (44 %)	<0.001
Yes			
Length of stay (days)			
	2 (2-2)	4 (4-5)	<0.001
Obstetric Complication			
No	30 (81 %) 7 (19 %)	7 (88 %) 1 (13 %)	0.67
Yes			
Foetal loss			
No	51 (96 %) 2 (4 %)	9 (100 %) 0 (0 %)	0.55
Yes			
Preterm labour			
No	33 (97 %) 1 (3 %)	6 (75 %) 2 (25 %)	0.029
Yes			
Apgar score			
1 min Apgar	8 (8-9)	8 (8-9)	0.66
5 min Apgar	9 (9-10)	9 (9-10)	0.65
Birth weight (grams)			
	3372 (538)	3320 (533)	0.81

Data are presented as mean (standard deviation) or median (interquartile range) for continuous variables, and N (%) for categorical variables.

No thromboembolic, septic or cardiac complications, Intensive Care Unit hospitalization, maternal mortality or fetal malfomations were recorded.

There were no differences in analysed variables between trimesters of pregnancy, except for obstetric complications and fetal loss (Table 2). Obstetric complications were more frequent in pregnant women operated during the first trimester, occurring in 45 % of patients. On the other hand, these were seen in 11 % of those operated in the second trimester and none were observed during the third trimester (p 0.02). Of the 44 patients followed up to the end of their pregnancy, eight had obstetric complications (18.2 %). There were 5 premature membrane ruptures, 1 intrauterine growth restriction, and two foetal losses, one preoperative and one verified after surgery, representing a postoperative foetal mortality of 1.6 %. Both occurred in pregnant women in the first trimester of pregnancy, with a statistically significant relationship between foetal mortality and the trimester in which the appendicitis occurred (p 0.04). One was found in the pre-surgical ultrasound and a uterine curettage was performed prior to laparoscopy. This patient was in her 8th week of pregnancy and presented an appendicular plastron with a cecal perforation, for which a right colectomy had to be performed. The other occurred in a pregnant woman on her 3rd week of gestation, who had a phlegmonous appendicitis. Spontaneous abortion occurred 10 days after laparoscopic appendectomy. This patient had already had a spontaneous abortion in her previous pregnancy.

**Table 2:** Variables according to the trimester of pregnancy at the time of surgery.

	TRIMESTER			p-value
VARIABLE	1	2	3	
Maternal age (years)				
	27 (6)	28 (6)	31 (4)	0.25
Prior abdominal surgery				
No	10 (67 %) 5 (33 %)	27 (79 %) 7 (21 %)	5 (71 %) 2 (29 %)	0.62
Yes				
White blood-cell count (cells/mm^3^)				
	14,586 (5817)	14,822 (3747)	16,164 (2192)	0.64
Murphy chronology				
No	6 (38 %) 10 (62 %)	17 (45 %) 21 (55 %)	2 (22 %) 7 (78 %)	0.45
Yes				
Rebound abdominal pain				
No	7 (44 %) 9 (56 %)	15 (39 %) 23 (61 %)	3 (33 %) 6 (67 %)	0.88
Yes				
Symptom duration prior to surgery (hours)				
	26	31	28	0.74
Type of Surgery				
Laparoscopy	1 (6 %) 14 (88 %) 1 (6 %)	3 (8 %) 35 (92 %) 0 (0 %)	0 (0 %) 9 (100 %) 0 (0 %)	0.44
Appendectomy				
Right Colectomy				
Intraoperative findings				
Normal appendix	2 (13 %) 11 (68 %) 2 (13 %) 1 (6 %)	3 (8 %) 28 (74 %) 6 (16 %) 1 (2 %)	1 (11 %) 5 (56 %) 2 (22 %) 1 (11 %)	0.9
Phlegmonous appendix				
Gangrenous appendix				
Appendicular plastron				
Conversion to open surgery				
No	15 (94 %) 1 (6 %)	38 (100 %) 0 (0 %)	8 (89 %) 1 (11 %)	0.17
Yes				
Peritonitis				
No	10 (62 %) 6 (38 %)	22 (58 %) 16 (42 %)	6 (67 %) 3 (33 %)	0.87
Yes				
Surgical time (minutes)				
	60 (60-68)	60 (45-60)	45 (45–100)	0.35
Tocolytic use				
No	16 (100 %) 0 (0 %)	34 (89 %) 4 (11 %)	8 (89 %) 1 (89 %)	0.4
Yes				
Intraoperative Complication				
No	16 (100 %) 0 (0 %)	37 (97 %) 1 (3 %)	9 (100 %) 0 (0 %)	0.72
Yes				
Postoperative Complication				
No	14 (88 %) 2 (12 %)	32 (84 %) 6 (16 %)	8 (89 %) 1 (11 %)	0.91
Yes				
Readmission				
No	16 (100 %) 0 (0 %)	35 (92 %) 3 (8 %)	8 (89 %) 1 (11 %)	0.45
Yes				
Length of Stay (days)				
	2 (1-4)	2 (2-3)	2 (2-3)	0.71
Obstetric Complication				
No	6 (55 %) 5 (45 %)	25 (89 %) 3 (11 %)	6 (100 %) 0 (0 %)	0.018
Yes				
Foetal loss				
No	13 (87 %) 2 (13 %)	38 (100 %) 0 (0 %)	9 (100 %) 0 (0 %)	0.039
Yes				
Preterm Labour				
No	7 (88 %) 1 (12 %)	26 (93 %) 2 (7 %)	6 (100 %) 0 (0 %)	0.67
Yes				
Birth weight (grams)				
	3132 (406)	3355 (555)	3660 (462)	0.2
Complete follow-up				
No Yes	6 (38 %) 10 (62 %)	10 (26 %) 28 (74 %)	3 (33 %) 6 (67 %)	0.7

Data are presented as mean (standard deviation) or median (interquartile range) for continuous variables, and N (%) for categorical variables.

Obstetric complications were independent of symptom duration, intraoperative findings, type of surgery performed or the occurrence of complications in the postoperative period.

Three preterm deliveries occurred, representing 6.8 % of the patients followed up to the end of pregnancy. No association with intraoperative findings or type of surgery performed was observed. However, there was an association between preterm delivery and the occurrence of postoperative complications (p 0.03) and obstetric complications (p 0.002).

All patients were followed 30 days after surgery and 69,8 (n = 44) were followed until the end of their pregnancy.

## 4 Discussion

Acute appendicitis is the most frequent cause of non-obstetric AAP, accounting for 70 % of AAP in our institution. There was a higher incidence of acute appendicitis in the second trimester, as reported in the literature [[16], [17], [18]]. However, unlike other authors [19,20], we did not find a higher incidence of appendicular perforation in the third trimester.

Pain in the right lower quadrant is reported to be found in most pregnant women with appendicitis, regardless of gestational age [[21], [22], [23]]. Except for the patient with situs inversus, who presented pain in the left lower quadrant, all women in our series presented pain in the right lower quadrant. Therefore, despite the intra-abdominal anatomical changes that occur with uterine growth, laxity of the abdominal wall musculature, physiological leucocytosis, and nausea and voiding common in pregnancy, the presence of pain in the right lower quadrant should motivate a high suspicion of acute appendicitis.

Surgery is the treatment of choice for appendicular AAP [15]. A gestational age limit has not been established for performing a laparoscopic appendectomy during pregnancy, although its difficulty could increase with increasing uterine size. Some have suggested the 28th week as the cut-off for using this approach [24]. Others consider laparoscopy an adequate diagnostic and therapeutic tool in any trimester, without increasing the maternal or foetal risk in advanced pregnancies [[25], [26], [27]]. Although there could be greater technical difficulty for laparoscopic surgery in the pregnant patient, we did not find difficulties to carry out the surgery, regardless of the trimester of pregnancy. We did not record injuries to the uterus or other abdominal or pelvic viscera, or present technical difficulties because of the uterus’ space occupation to motivate conversion to open surgery. We performed 9 surgeries after the 28th week of pregnancy, and found no differences in surgical times, need for conversion, type of surgery, or surgical complications between trimesters. Taking this into account, laparoscopy would be a useful tool, providing the benefits of minimally invasive surgery, in all cases.

The abdominal cavity access technique in pregnant patients is controversial. Complications have been described with any entry technique [[28], [29], [30], [31]]. In our series, open access with Hasson was routinely used and no uterine, vascular or visceral lesions occurred. Current SAGES recommendation is that either Veress or Hasson techniques can be used safely and effectively, as long as the initial access site to the abdomen is made taking into account the height of the uterine fundus, and the abdominal wall is pulled upwards during entry [15].

The rate of negative laparoscopy varies considerably between studies (Table 3). The higher incidence during pregnancy is probably due to diagnostic difficulty and attempts to prevent the progression towards perforation, due to the relationship between this and maternal/foetal morbidity and mortality [[32], [33], [34], [35], [36]]. Some even propose laparoscopy as an initial diagnostic tool, allowing optimization of diagnostic precision, and being able to treat the pathology without delay [37,38]. However, in a systematic review carried out by Walsh et al. [39], a similar foetal death rate was found between those operated with acute appendicitis and those in which a negative laparoscopy was performed, which would indicate that it is important to achieve a balance between avoiding diagnostic delay and the risks of unnecessary surgery. We had 6 patients in which a normal appendix was found, representing an incidence of 9.5 %, and we did not find surgical, obstetric complications or foetal mortality in this group.

**Table 3:** Results of laparoscopic treatment of acute appendicitis in pregnant women.

Author	Year	N	Postoperative Complication	Obstetric Complication	Preterm Labour	Foetal loss	Negative Laparoscopy
Machado [57]	2009	20^$^	10 %	10 %	5 %	5 %	25 %
Ito [58]	2012	87^&^	5,8 %	10,3 %	23 %	5,8 %	36 %
Lemieux et al. [59]	2008	45	8 %	NR	18,9 %	0 %	33 %
Sadot [60]	2010	48	4 %	2 %	29 %	2 %	27 %
Rollins[10]	2004	30^+^	3,3 %	3,3 %	21,4 %	NR	41 %
Affleck [61]	1999	23	NR	NR	15,8 %	0 %	26 %
Halkic [62]	2006	11	0 %	NR	0 %	0 %	9,1 %
Halvorsen [44]	1994	16	18,75 %	NR	12,5 %	6,25 %	25 %
McGory[34]	2007	454	7 %	NR	<1 %	7 %	27 %
Vaseileiou [63]	2019	35	8,6 %	NR	NR	NR	8,6 %
Cheng [64]	2014	128	NR	NR	5,5 %	5,5 %	NR
Erekson [65]	2012	550	2,9 %	NR	NR	NR	NR
Butte et al. [66]	2006	45	28,3 %	17,8 %	15,6 %	2,2 %	11,1 %
Kirshtein et al. [67]	2009	23	0 %	27 %	NR	4,3 %	13 %
Schreiber [68]	1990	6	0 %	NR	NR	0 %	33 %
Corneille[69]	2010	9	NR	11,1 %	11,1 %	0 %	NR
Chung[70]	2013	22	4,5 %	NR	9,1 %	0 %	9 %
Peled [71]	2014	26	3,8 %	NR	19,2 %	3,8 %	19,2 %
Carver [72]	2005	17	5,9 %	NR	0 %	11,8 %	NR
Palianivelu [73]	2007	7	14,2 %	0 %	0 %	0 %	0 %
Moreno Sanz [74]	2007	6	0 %	16,7 %	16,7 %	0 %	NR
Schmidt [75]	2007	15	13,3 %	NR	13,3 %	0 %	NR
Wu [76]	2005	11	9,1 %	9,1 %	NR	9,1 %	0 %
Rizzo [77]	2003	4	0 %	NR	0 %	0 %	NR
Lyass [78]	2001	11	9,1 %	9,1 %	0 %	0 %	9,1 %
dePerrot [79]	2000	6	0 %	33,3 %	16,7 %	33,3 %	50 %
Andreoli [80]	1999	5	0 %	20 %	0 %	0 %	60 %
Lemaire [81]	1997	4	0 %	25 %	0 %	0 %	NR
Gurbuz [82]	1997	9^@^	NR	NR	0 %	0 %	22,2 %
Lautsen [83]	2016	19	5,3 %	NR	15,8 %	0 %	15,8 %
Chwat (current series)	2021	63	14,2 %	18,2 %^#^	6,8 %	1,6 %	9,5 %

When in the same series there are data from conventional appendectomies, and the author has not discriminated between groups when reporting results, they are described together with the laparoscopy data. If the author discriminated between groups, only the results of the laparoscopic approach are reported.

NR = not reported

$ Six of them were conventional appendectomies.

+ Two of them were conventional appendectomies.

# The 2 foetal losses are included within obstetric complications.

& Does not clarify if laparoscopic or conventional approach was used.

@ Four of them were conventional appendectomies.

As described by other authors [40,41,42,43], a statistically significant relationship was found between the duration symptoms prior to surgery and the intraoperative findings, noting three times more appendiceal perforation when symptom duration was greater than or equal to 48 h. Likewise, the only independent variable that was statistically associated with the development of postoperative maternal complications was symptom duration greater than or equal to 48 h. Therefore, prompt diagnosis and treatment are crucial.

Some authors recommend systematic tocolytic use [44,45]. However, following the obstetric indications of our institution, and in agreement with other authors [46,47], we only used tocolytics in 7.9 % of the patients, given the existence of uterine dynamics and risk of premature birth.

We found 14.3 % of postoperative complications. However, only two complications were major complications: one patient with intra-abdominal abscesses that led to a reoperation and another with an intra-abdominal abscess secondary to an appendicular stump fistula that did not require reoperation (3.2 %). The report of postoperative complications in the literature is heterogeneous since it is limited, for the most part, to what authors define as “major complications”, and there is no consensus for the definition of this type of complications. The same is true for obstetric and perinatological complications. The Clavien-Dindo Classification [48] is a widely used tool for evaluating and reporting postoperative complications in surgery. However, it consider the particular situation of the pregnant woman, considering simultaneously complications inherent to the foetus and the mother. The absence of a tool for an objective registry of complications could explain the variability of reported outcomes (Table 3), and therefore the inability to reliably collate the results from the literature.

A higher risk of delayed intrauterine growth, prematurity and low birth weight are described in pregnant women undergoing appendectomy, regardless of the approach used, type of appendicitis, the anaesthesia or the surgical approach [49]. In our experience, we recorded three preterm deliveries and one case of intrauterine growth restriction. None of the observed obstetric complications was correlated with the time from symptom onset to surgery. The weight of term new-borns of women operated on for acute appendicitis during pregnancy was similar to that of those born to non-operated mothers.

Systematic reviews and meta-analyses of laparoscopic appendectomy during pregnancy have reported a benefit of laparoscopy due to a lower incidence of complications and shorter hospital stay, but a higher risk of foetal loss with laparoscopy than with conventional surgery [50,51]. In our cohort, there was one postoperative foetal death, representing a low foetal mortality rate when compared to other studies that present foetal mortality ranging from 0 % −33.3 % (Table 3).

The reported foetal death rate is between 3 and 5% in uncomplicated appendicitis, and 20–36 % in cases of perforated appendicitis [52,53]. However, we did not find an association between foetal death and appendicular perforation. Unlike that reported by Babler [54], we did not find an association between foetal loss and the delay from the onset of symptoms to surgical resolution. Additionally, we did not find an association between foetal death and intraoperative findings or the occurrence of complications in the postoperative period.

Laparoscopic procedures in the first trimester have been associated with a higher probability of foetal death than in other trimesters [55]. However, the incidence of baseline foetal death, not related to surgery, is also higher during the first trimester [56]. We found an association between foetal mortality and the trimester in which the surgery was performed. Both cases reported in our series occurred in patients in the first trimester of pregnancy, one of them in the 8th week and the other in the 3rd week of gestation. It should be noted that the postoperative foetal death occurred in a patient who had a history of a previous spontaneous abortion, so foetal death might not be related to the surgical procedure.

The analysed literature includes clinical cases and retrospective series such as the one presented by our group. Due to its retrospective nature, this study has limitations, including the lack of information regarding the evolution of pregnancies in those patients who attended their deliveries outside our institution. This did not allow follow-up data up to the end of pregnancy in this group. However, all patients were followed for the first thirty days after surgery, so the development of postoperative complications was fully documented.

## 5 Conclusion

Surgical morbidity of acute appendicitis in pregnant women is linked to delay in diagnosis and treatment of the inflammatory condition.

Laparoscopic appendectomy in pregnant women appears to be an effective technique in all trimesters of pregnancy, providing the benefits of minimally invasive surgery; provided that the specific recommendations for this type of patient are respected. However, it is not exempt from postoperative complications, obstetric complications, risk of preterm delivery and foetal loss.

It is necessary to standardize the definitions of “complication” in order to collate reliably the outcomes presented in the literature.

## Provenance and peer review

Not commissioned, externally peer-reviewed.

## Declaration of competing interest

None.

## Research registration unique identifying number (UIN)

Registry used: www.clinicaltrials.gov

ClinicalTrials.gov Identifier: NCT04753502

https://register.clinicaltrials.gov/prs/app/action/SelectProtocol?sid = S000AMXQ&selectaction = Edit&uid = U0005HST&ts = 5&cx = 8tszrc.

## Data statement

Construction and analysis of the database and study protocol were authorized under N° B20-010 and P20-070, respectively, by the Institutional Evaluation Committee of the Austral University Hospital, Buenos Aires, Argentina.

Data will be made available on request.
